# Coping with Stress and Types of Burnout: Explanatory Power of Different Coping Strategies

**DOI:** 10.1371/journal.pone.0089090

**Published:** 2014-02-13

**Authors:** Jesus Montero-Marin, Javier Prado-Abril, Marcelo Marcos Piva Demarzo, Santiago Gascon, Javier García-Campayo

**Affiliations:** 1 Faculty of Health Sciences and Sports, University of Zaragoza, Huesca, Spain; 2 Department of Psychiatry, University of Zaragoza, Zaragoza, Spain; 3 Psychiatry Service, Miguel Servet University Hospital, Zaragoza, Spain; 4 Department of Preventive Medicine, Universidade Federal de São Paulo (UNIFESP), São Paulo, Brasil; 5 Department of Psychology, University of Zaragoza, Teruel, Spain; 6 REDIAPP “Red de Investigación en Actividades Preventivas y Promoción de la Salud” (Research Network on Preventative Activities and Health Promotion), Zaragoza, Spain; University of St Andrews, United Kingdom

## Abstract

**Background:**

Burnout occurs when professionals use ineffective coping strategies to try to protect themselves from work-related stress. The dimensions of ‘overload’, ‘lack of development’ and ‘neglect’, belonging to the ‘frenetic’, ‘under-challenged’ and ‘worn-out’ subtypes, respectively, comprise a brief typological definition of burnout. The aim of the present study was to estimate the explanatory power of the different coping strategies on the development of burnout subtypes.

**Methods:**

This was a cross-sectional survey with a random sample of university employees, stratified by occupation (n = 429). Multivariate linear regression models were constructed between the ‘Burnout Clinical Subtypes Questionnaire’, with its three dimensions –overload, lack of development and neglect– as dependent variables, and the ‘Coping Orientation for Problem Experiences’, with its fifteen dimensions, as independent variables. Adjusted multiple determination coefficients and beta coefficients were calculated to evaluate and compare the explanatory capacity of the different coping strategies.

**Results:**

The ‘Coping Orientation for Problem Experiences’ subscales together explained 15% of the ‘overload’ (p<0.001), 9% of the ‘lack of development’ (p<0.001), and 21% of the ‘neglect’ (p<0.001). ‘Overload’ was mainly explained by ‘venting of emotions’ (Beta = 0.34; p<0.001); ‘lack of development’ by ‘cognitive avoidance’ (Beta = 0.21; p<0.001); and ‘neglect’ by ‘behavioural disengagement’ (Beta = 0.40; p<0.001). Other interesting associations were observed.

**Conclusions:**

These findings further our understanding of the way in which the effectiveness of interventions for burnout may be improved, by influencing new treatments and preventive programmes using features of the strategies for handling stress in the workplace.

## Introduction

Policies and interventions to promote mental health should be designed to effectively involve the work environment and process as a key arena for action [1]. The majority of people in developed and developing countries now live in cities and are formally or informally linked to workplaces where most of their productive lives are spent [2]. Studies have shown the importance of work stressors both in the generation and prevention of mental disorders [3], but there is still a lack of policies and interventions that effectively improve workers’ mental health and prevent disorders. Interestingly, even among mental health workers, work-related mental disorders are highly prevalent [4]. Thus, work environments and processes are key elements in public health.

Burnout syndrome is an important work-related disorder of psychosocial origin, caused when stressful working conditions are endured. Its presence has been associated with a worsened self-perception of health and a large amount of somatic comorbidity [5]. Burnout has traditionally been described as a relatively uniform entity in all individuals, with more or less consistent aetiology and symptoms [6]. According to the classical definition, this syndrome includes the dimensions of exhaustion, cynicism and professional inefficacy [7], [8]. ‘Exhaustion’ is the feeling of not being able to offer any more of oneself at an emotional level; ‘cynicism’ represents a distant attitude towards work, those served by it, and colleagues; and ‘inefficacy’ is the feeling of not performing tasks adequately or being incompetent at work. These dimensions are strongly associated with each other, providing a unitary although three-dimensional definition of burnout [9].

Nevertheless, different burnout types have been proposed, according to the degree of dedication at work [10]. The ‘frenetic’ burnout type works increasingly harder, to the point of exhaustion, in search of success, and presents involvement, ambition and overload. The ‘under-challenged’ type has to cope with monotonous and unstimulating conditions that fail to provide satisfaction and feels indifference, boredom and lack of personal development. The ‘worn-out’ type gives up when faced with stress or the absence of gratification and shows lack of control, lack of acknowledgement and neglect [11], [12]. The dimensions of overload, lack of development and neglect, belonging to the frenetic, under-challenged and worn-out subtypes, respectively, comprise a definition of burnout that comes close to the standard perspective [9], [13]. ‘Overload’ refers to individuals’ feeling of risking health and personal life in the pursuit of good results and is significantly associated with exhaustion; ‘lack of development’ refers to the absence of personal growth experiences for individuals together with their desire to take on other jobs where they can better develop their skills and is markedly associated with cynicism; ‘neglect’ refers to individuals’ disregard as a response to any difficulty and is strongly associated with inefficacy [13], [14]. While approaching the standard definition, the dimensions referred to in the typological model show little relation to each other, which allows a differential characterisation of the syndrome to be made by means of clinical profiles [13].

In general, ‘burnout’ is a subject’s response to chronic work-related stress and is an attempt to adapt to or protect oneself from it [15]. Stress has been defined as the result of a relationship with the environment that the person appraises as significant for his or her well-being, and in which demands tax or exceed available coping resources. Coping is defined as cognitive and behavioural efforts to manage specific internal and/or external demands that are appraised as taxing or exceeding the person’s resources [10], [16]. A person will be psychologically vulnerable to a particular situation if he or she does not possess sufficient coping resources to handle it adequately and places considerable importance on the threat implicit in the consequences of this inadequate handling. There are different general trends in coping with stress, such as cognitive or behavioural coping, cognitive or behavioural avoidance, emotion-focused coping or substance use [17]–[19]. From this perspective, burnout may be observed as a progressively developed condition resulting from the use of the ineffective coping strategies with which professionals try to protect themselves from work-related stress situations [20].

There is an accumulation of evidence linking coping styles with stress and burnout. At first, coping style was studied as a relatively stable characteristic of the person, regardless of the nature of the task or situation, showing that certain inflexible coping styles could be associated with higher levels of stress [21], [22]. Subsequently, the emphasis was placed on the relationship between the coping style and the situation [16]. Early research seemed to support the idea that problem-focused coping was a better strategy than emotion-focused coping for stress management. However, it was later found that there were sub-factors that did not allow the application of such a general conclusion [23]. Problem-focused coping is not an appropriate strategy to address stress if the situation is uncontrollable or chronic [24], as it could lead, in this case, to a progressive process of behavioural disengagement [25]. Emotional coping has been noted to be detrimental if it involves distancing, avoidance or denial regarding the situation but is an effective strategy if it involves a positive reappraisal [26], [27]. In the long term, the key factor for developing the burnout syndrome seems to be the degree of passivity that the subject acquires [19], [28], [29].

So far, possible relationships between burnout types and coping strategies have not been explored. A better knowledge of the coping strategies associated with each burnout profile could promote the development of specific treatments and preventive programmes for the syndrome that might potentially be more effective [26]. In this context, the aim of this work was to estimate the explanatory power of the different styles of coping with stress on the development of different burnout subtypes, evaluating the contribution of specific coping strategies. In general terms, the hypotheses were established according to the degree of dedication at work shown by the different burnout subtypes. The frenetic burnout subtype is a highly dedicated profile, which means that the related overload could be associated with active coping strategies, such as those included in problem-focused coping. The under-challenged burnout subtype is a profile characterised by an intermediate dedication to work, meaning that the related lack of development could be associated with avoidance coping strategies. The worn-out burnout subtype is a profile characterised by a low level of dedication, meaning that the associated neglect could be due to a behavioural impairment related to the use of disengagement strategies. In essence, this grading of the levels of dedication could be pointing to different stages in the longitudinal development of the syndrome. Different coping strategies for stress could be contributing to each of these [10], [12].

## Methods

### Study design

We used a cross-sectional survey design. Participants gave informed consent and then completed an online self-assessment survey.

### Participants

The study population consisted of all employees of the University of Zaragoza, Spain, who were working in January 2008 (N = 5,493), comprising a multi-occupational group that included jobs of differing nature and complexity. These workers form a population that is at risk of developing burnout, as they consist of professionals working face-to-face with other people [15]. The required sample size was calculated to be able to make estimates with a 95% confidence level, with a 3.5% margin of error, and assuming an 18% prevalence of burnout [30]; thus, the sample size needed was 427 participants. Given that the response rate for previous web-mail surveys had been approximately 27% [31], [32], 1,600 people were selected from an alphabetical list of the entire workforce by means of random stratified sampling with proportional allocation depending on occupation (58% teaching and research staff –‘TRS’, 33% administration and service personnel –‘ASP’, and 9% grant holders –‘GRH’. GRH refers to ‘undergraduate’ or ‘postgraduate’ students, employed by the university to reinforce different types of services, and to ‘pre-doctoral’ or ‘post-doctoral’ students, both postgraduates, specifically employed to perform research project-related tasks. Sample size calculation and random sampling were performed using Epidat 3.1 software.

### Procedure and ethics statement

In April 2008, an e-mail was sent to the selected individuals explaining the aims of the research, to whom it was directed, the voluntary nature of participation, the potential benefits and risks, and data confidentiality. This message contained a link to the online questionnaire and access passwords for participants to complete the questionnaire, after providing informed consent. Participants had to mark the acceptance of the conditions imposed by the consent form in order to activate the access passwords. Upon completion of the survey, all participants received an anonymous report with an explanation of their results, as gratitude. The project was approved by the Clinical Research Ethics Committee of Aragon, Spain.

### Measurements

#### Sociodemographic and occupational characteristics

Participants were first asked a set of questions dealing with sociodemographic and occupational characteristics, including the following: age, sex, relationship status (‘yes’ or ‘no’ to being in a stable relationship), level of education (‘secondary or lower’, ‘university degree’, ‘doctorate’), occupation type (‘TRS’, ‘ASP’, ‘GRH’ – and within this group ‘undergraduate’, ‘post-graduate’, ‘pre-doctoral’ and ‘post-doctoral’), years of service (‘<4’, ‘4–16’, ‘>16’ – according to the sociodemographic characterisation of the burnout profiles [11], [33]), contract duration (‘permanent’ vs ‘temporary’), contract type (‘full-time’ vs ‘part-time’), whether they had taken sick leave in the previous year (‘yes’ vs ‘no’), and the number of sick leave days taken.

#### Burnout subtypes

Participants were asked to complete the ‘Burnout Clinical Subtypes Questionnaire’ (BCSQ-12) in its Spanish language version [13]. This questionnaire consists of 12 items, evenly distributed into 3 dimensions (comprising 4 items in each). The ‘frenetic’ subtype is represented by the ‘overload’ dimension (e.g., “I overlook my own needs to fulfil work demands”); the ‘under-challenged’ subtype by the ‘lack of development’ dimension (e.g., “My work doesn’t offer me opportunities to develop my abilities”); and the ‘worn-out’ subtype by the ‘neglect’ dimension (e.g., “When things at work don’t turn out as well as they should, I stop trying”). Participants had to indicate the degree to which they agreed with each of the statements presented according to a Likert-type scale with 7 response options, scored from 1 (‘totally disagree’) to 7 (‘totally agree’). Each of the dimensions received a score, which is presented as a sum of its constituent items divided by the number of items (scaled score). The factorial validity of the BCSQ-12 presents consistent results in the study population, with α≥0.80 reliability for each of the constituent dimensions and good power for explaining the burnout standard measures [9], [13].

#### Coping strategies

Participants were then asked to complete the Spanish version of the ‘Coping Orientation for Problem Experiences’ (COPE) [17], [18]. This instrument, widely used for the evaluation of stress-coping strategies, incorporates 60 items distributed into 15 scales that show the behaviour implemented in the coping process to address stress. In the Spanish version, these scales are ‘social support’, ‘religion’, ‘humour’, ‘substance use’, ‘planning’, ‘behavioural disengagement’, ‘venting of emotions’, ‘acceptance’, ‘denial’, ‘restraint’, ‘focus on solving situations’, ‘personal growth’, ‘positive reinterpretation’, ‘distracting activities’ and ‘cognitive avoidance’. Participants had to indicate the degree to which they agreed with each of the items according to a Likert-type scale with four response options, scored from 1 (‘I don’t usually do this at all’) to 4 (‘I usually do this a lot’). The score from each dimension was presented as a sum of its constituent items divided by the number of items (scaled score). The instrument presents adequate psychometric properties in the original and adapted versions; it shows good levels of internal consistency and test-retest reliability; and it has been specifically used in other studies with employees of Spanish universities [17], [18], [34].

### Data analysis

A descriptive analysis of participants' sociodemographic and occupational characteristics was made using means and percentages according to the nature of the variables.

The explanatory power of the coping strategies in relation to the burnout types was assessed by constructing multiple linear regression models. For this purpose, the BCSQ-12 subscales overload, lack of development and neglect were considered dependent variables, while the dimensions of the COPE were considered independent variables, so that three models were constructed. The possible influence of the sociodemographic variables were controlled, and they were included in each model. The predictive capacity of the models was examined by the significance of the F value associated with the regression by means of analysis of variance. Multiple correlation coefficients (R_y.123_) were calculated to quantify the degree of association between each dependent variable and the independent variables taken as a set. Multiple determination coefficients (R^2^_y.123_) and adjusted multiple determination coefficients (adj-R^2^_y.123_), were also calculated to evaluate the explanatory capacity of the coping strategies [35], [36].

The ‘raw’ relationship of each independent variable with each dependent variable was calculated by applying Pearson's r correlation coefficient. The individual contribution of the independent variables in each multivariate model was estimated by means of the calculation of the standardised slope coefficients (Beta). Partial correlation coefficients (R_y3.12_) were calculated, indicating the correlation between two variables when the effect of the other variables included in the equation was removed. Semi-partial correlation coefficients (R_y(3.12)_) were also calculated, the square of which showed the increase in the coefficient of determination after including a specific variable in a model, partialising the influence of the other included variables. The Wald test was used to evaluate the statistical significance of the contribution of each variable to each model. Tolerance (T) values were calculated to rule out possible collinearity problems. The Kolmogorov-Smirnov (KS) test was used to determine whether the conditional distribution of the residuals met the assumption of normality. Finally, it was confirmed that the Durbin-Watson values (DW) approached a value ≈2.00 to rule out autocorrelation problems in the errors [35], [36].

All of the tests were bilateral and were performed with a significance level of α<0.05. Data analysis was conducted with the SPSS-15 statistical software package.

## Results

In order to adhere to standards for data availability, all materials used to produce the results in this paper will be made available upon request. This includes [37]: 1.- The list of documents and data files that are needed in order for replication to be possible, 2.- A detailed list of what will be provided by the authors, and 3.- What steps, and in what sequence, the interested researchers need to take in order for this data to be made available. In addition, the authors will post these materials on the group's website [38].

### Sample characteristics

A total of 429 respondents were included in the study, representing a response rate (RR) of 26.8%. The RRs by occupation were distributed as follows: 21.6% ‘TRS’, 31.1% ‘ASP’, 43.1% ‘GRH’ (χ^2^ = 37.44; df = 2; p<0.001). The mean age of participants was 40.10 years (SD = 9.98), with 43.9% males. The majority (78.4%) were in a stable relationship and 13.6% had achieved secondary or lower schooling; 50.2% had university degrees and 36.2% held doctorates. In terms of job position, 46.9% were ‘TRS’; 38.5% were ‘ASP’; and 14.6% were ‘GRH’ (11.3% ‘undergraduate’, 6.4% ‘postgraduate’, 74.2% ‘pre-doctoral’ and 8.1% ‘post-doctoral’). In terms of length of employment, 25.5% had been working at the university for ‘less than 4 years’, with 41.1% working ‘between 4 and 16 years’ and 33.4% for ‘more than 16 years’. In total, 58.7% were permanent employees and the majority (88.3%) worked full time. During the previous year, 29.8% of the participants had taken sick leave. The mean sick leave days for those who had taken them was 24.88 days (SD = 66.22).

### Descriptives and raw correlations

The BCSQ-12 subscales showed the following descriptive results: ‘overload’ Mean = 3.41 (SD = 1.53), ‘lack of development’ Mean = 3.14 (SD = 1.68) and ‘neglect’ Mean = 2.20 (SD = 1.06). Table 1 shows the descriptives of the COPE subscales. Table 1 also presents the r values for the raw correlation between the subscales. As can be observed, all of the BCSQ-12 dimensions showed significant associations with some of the coping strategies.

**Table 1:** Descriptives and raw correlations for the BCSQ-12 and COPE subscales.

Coping strategies	Mean	SD	Overload	Lack of development	Neglect
Social support	2.50	0.68	0.12*	−0.08	−0.08
Religion	1.37	0.69	0.12*	0.08	0.01
Humour	2.07	0.76	0.02	<0.01	−0.04
Substance use	1.12	0.40	0.13**	0.08	0.11**
Planning	2.67	0.60	0.14**	−0.13**	−0.22***
Behavioural disengagement	1.52	0.64	−0.02	0.23***	0.45***
Venting of emotions	2.28	0.66	0.34***	0.15**	0.18***
Acceptance	2.51	0.64	−0.02	−0.06	−0.02
Denial	1.60	0.41	0.12*	0.15**	0.11*
Restraint	2.35	0.50	0.08	0.03	0.10*
Focus on solving situations	2.37	0.61	0.24***	−0.07	−0.09
Personal growth	2.98	0.72	0.04	−0.06	−0.18***
Positive reinterpretation	2.61	0.61	0.11*	−0.05	−0.11*
Distracting activities	2.23	0.50	0.10*	0.03	0.02
Cognitive avoidance	1.65	0.54	0.08	0.29***	0.27***

*** p<0.001; ** p<0.01; * p<0.05 (bilateral).

### Regression models

As observed in Tables 2, 3 and 4, the explanatory power of all models was reasonable. The most explained burnout dimension was ‘neglect’ (21%), whilst the least explained burnout dimension was ‘lack of development’ (9%), with ‘overload’ in the middle (15%). The fit of the multivariate linear regression models, which was evaluated using the variance analysis, was good in all cases (p<0.001), with adequate standard error values. DW values were all appropriate, ruling out self-correlation problems in the errors. Residual distribution was normal in all cases, making it possible to accept the basic assumptions needed to go ahead with the regression. The T values of variables were high, meaning that they were models without redundant variables for information purposes. As can be observed, the standard errors from slopes were low (<0.25). The main coping strategy that contributed to explaining ‘overload’ was ‘venting of emotions’ (Beta = 0.34; p<0.001). ‘Cognitive avoidance’ was the main coping strategy explaining ‘lack of development’ (Beta = 0.21; p<0.001). ‘Neglect’ was mainly explained by ‘behavioural disengagement’ (Beta = 0.40; p<0.001). Not all intercepts were significant.

**Table 2:** Regression coefficients for the COPE with regard to the ‘Overload’ dimension of the BCSQ-12.

Coping strategies	R_y.123_	R^2^_y.123_	adj-R^2^_y.123_	F (df_1_/df_2_) p^a^	Se	DW	p^b^
	0.44	0.19	0.15	5.24 (18/404) <0.001	1.41	1.92	0.767
	R_y3.12_	R_y(3.12)_	T	B (95% CI)	Se	Beta	p^c^
*Intercept*				0.17 (−1.34–1.67)	0.76		0.828
Social support	−0.09	−0.08	0.63	−0.24 (−0.49–0.02)	0.13	−0.10	0.068
Religion	0.10	0.09	0.89	0.22 (0.01–0.43)	0.11	0.10	0.040*
Humour	0.04	0.03	0.76	0.07 (−0.13–0.28)	0.10	0.04	0.487
Substance use	0.05	0.05	0.81	0.19 (−0.18–0.57)	0.19	0.05	0.316
Planning	0.03	0.02	0.46	0.09 (−0.25–0.42)	0.17	0.03	0.605
Behavioural disengagement	−0.02	−0.02	0.60	−0.05 (−0.32–0.22)	0.14	−0.02	0.728
Venting of emotions	0.30	0.29	0.72	0.78 (0.54–1.02)	0.12	0.34	<0.001*
Acceptance	−0.04	−0.04	0.68	−0.12 (−0.37–0.14)	0.13	−0.05	0.377
Denial	0.04	0.04	0.61	0.17 (−0.25–0.59)	0.21	0.05	0.421
Restraint	0.01	0.01	0.61	0.04 (−0.30–0.38)	0.17	0.01	0.827
Focus on solving situations	0.11	0.10	0.55	0.33 (0.04–0.63)	0.15	0.13	0.029*
Personal growth	0.01	0.01	0.51	0.04 (−0.22–0.30)	0.13	0.02	0.780
Positive reinterpretation	0.05	0.05	0.52	0.17 (−0.14–0.48)	0.16	0.07	0.290
Distracting activities	−0.01	−0.01	0.62	−0.04 (−0.38–0.31)	0.18	−0.01	0.826
Cognitive avoidance	−0.02	−0.02	0.68	−0.07 (−0.37–0.24)	0.15	−0.02	0.664

R_y.123_ = multiple correlation coefficient. R^2^_y.123_ = coefficient of multiple determination. adj-R^2^_y.123_ = adjusted coefficient of multiple determination. p^a^ = p value for variance analysis associated with the regression. Se = standard error. DW = Dubin-Watson value. p^b^ = p value for K-S test for normality contrast on residuals. R_y3.12_ = partial correlation coefficient. R_y(3.12)_ = semi-partial correlation coefficient. T = tolerance value. B = regression slope. CI = confidence interval. Beta = standardised slope. p^c^ = p value of Wald test result. * = significant value (p<0.05).

**Table 3:** Regression coefficients for the COPE with regard to the ‘Lack of development’ dimension of the BCSQ-12.

Coping strategies	R_y.123_	R^2^_y.123_	adj-R^2^_y.123_	F (df_1_/df_2_) p^a^	Se	DW	p^b^
	0.36	0.13	0.09	3.36 (18/404) <0.001	1.60	2.04	0.060
	R_y3.12_	R_y(3.12)_	T	B (95% CI)	Se	Beta	p^c^
*Intercept*				2.09 (0.38–3.79)	0.87		0.016
Social support	−0.09	−0.08	0.63	−0.25 (−0.54–0.04)	0.15	−0.10	0.086
Religion	0.07	0.07	0.89	0.18 (−0.06–0.41)	0.12	0.07	0.140
Humour	0.02	0.01	0.76	0.04 (−0.20–0.27)	0.12	0.02	0.763
Substance use	−0.04	−0.04	0.81	−0.18 (−0.60–0.25)	0.22	−0.04	0.415
Planning	−0.04	−0.04	0.46	−0.17 (−0.55–0.21)	0.19	−0.06	0.387
Behavioural disengagement	0.10	0.10	0.60	0.32 (0.01–0.63)	0.16	0.12	0.041*
Venting of emotions	0.11	0.10	0.72	0.30 (0.03–0.57)	0.14	0.12	0.032*
Acceptance	−0.09	−0.08	0.68	−0.26 (−0.55–0.04)	0.15	−0.10	0.085
Denial	0.03	0.03	0.61	0.16 (−0.31–0.63)	0.24	0.04	0.501
Restraint	−0.02	−0.02	0.61	−0.08 (−0.47–0.31)	0.20	−0.02	0.695
Focus on solving situations	0.03	0.02	0.55	0.09 (−0.25–0.43)	0.17	0.03	0.606
Personal growth	0.05	0.05	0.51	0.15 (−0.15–0.44)	0.15	0.06	0.328
Positive reinterpretation	0.05	0.04	0.52	0.16 (−0.19–0.51)	0.18	0.06	0.363
Distracting activities	−0.04	−0.04	0.62	−0.16 (−0.55–0.23)	0.20	−0.05	0.427
Cognitive avoidance	0.19	0.18	0.68	0.66 (0.32–1.00)	0.18	0.21	<0.001*

R_y.123_ = multiple correlation coefficient. R^2^_y.123_ = coefficient of multiple determination. adj-R^2^_y.123_ = adjusted coefficient of multiple determination. p^a^ = p value for variance analysis associated with the regression. Se = standard error. DW = Dubin-Watson value. p^b^ = p value for K-S test for normality contrast on residuals. R_y3.12_ = partial correlation coefficient. R_y(3.12)_ = semi-partial correlation coefficient. T = tolerance value. B = regression slope. CI = confidence interval. Beta = standardised slope. p^c^ = p value of Wald test result. * = significant value (p<0.05).

**Table 4:** Regression coefficients for the COPE with regard to the ‘Neglect’ dimension of the BCSQ-12.

Coping strategies	R_y.123_	R^2^_y.123_	adj-R^2^_y.123_	F (df_1_/df_2_) p^a^	Se	DW	p^b^
	0.50	0.25	0.21	7.31 (18/404) <0.001	0.93	2.00	0.110
	R_y3.12_	R_y(3.12)_	T	B (95% CI)	Se	Beta	p^c^
*Intercept*				1.34 (0.35–2.34)	0.51		0.008
Social support	−0.04	−0.04	0.63	−0.07 (−0.24–0.10)	0.09	−0.05	0.395
Religion	<−0.01	<−0.01	0.89	<−0.01 (−0.14–0.14)	0.07	−0.00	0.964
Humour	−0.02	−0.02	0.76	−0.03 (−0.17–0.10)	0.07	−0.02	0.635
Substance use	−0.01	−0.01	0.81	−0.01 (−0.26–0.24)	0.13	−0.01	0.921
Planning	−0.10	−0.08	0.46	−0.22 (−0.44–0.01)	0.11	−0.12	0.057
Behavioural disengagement	0.33	0.31	0.60	0.65 (0.47–0.83)	0.09	0.40	<0.001*
Venting of emotions	0.09	0.08	0.72	0.15 (−0.01–0.31)	0.08	0.10	0.060
Acceptance	−0.09	−0.07	0.68	−0.15 (−0.32–0.02)	0.09	−0.09	0.087
Denial	−0.08	−0.07	0.61	−0.21 (−0.49–0.06)	0.14	−0.08	0.128
Restraint	0.09	0.07	0.61	0.20 (−0.03–0.42)	0.12	0.10	0.086
Focus on solving situations	0.08	0.07	0.55	0.16 (−0.04–0.36)	0.10	0.09	0.117
Personal growth	−0.07	−0.06	0.51	−0.12 (−0.29–0.06)	0.09	−0.08	0.184
Positive reinterpretation	0.08	0.07	0.52	0.18 (−0.03–0.38)	0.10	0.10	0.094
Distracting activities	0.02	0.02	0.62	0.05 (−0.18–0.28)	0.12	0.02	0.691
Cognitive avoidance	0.08	0.07	0.68	0.17 (−0.03–0.37)	0.10	0.09	0.094

R_y.123_ = multiple correlation coefficient. R^2^_y.123_ = coefficient of multiple determination. adj-R^2^_y.123_ = adjusted coefficient of multiple determination. p^a^ = p value for variance analysis associated with the regression. Se = standard error. DW = Dubin-Watson value. p^b^ = p value for K-S test for normality contrast on residuals. R_y3.12_ = partial correlation coefficient. R_y(3.12)_ = semi-partial correlation coefficient. T = tolerance value. B = regression slope. CI = confidence interval. Beta = standardised slope. p^c^ = p value of Wald test result. * = significant value (p<0.05).

## Discussion

This is the first study that has evaluated the explanatory power of different coping strategies in relation to the brief typological definition of burnout syndrome [13]. Other works have indicated the relevance of coping on burnout syndrome as classically defined [39], but not on the subtypes. Multiple regression analysis showed that the dimensions of the BCSQ-12 were significantly explained by the coping strategies. Overall, the starting hypotheses were confirmed: overload was explained by the focus on the solving of situations, although it was also explained by religion and mainly by venting of emotions; lack of development was explained mainly by cognitive avoidance, although it was also explained by venting of emotions and behavioural disengagement; and neglect was only explained by behavioural disengagement, which is consistent with the general proposals of a previous study [33]. Other interesting associations were observed that might be consistent with the idea that the development of the syndrome would correspond with the burnout types as stages [10], [12]. These findings may be relevant to improve the effectiveness of current interventions on burnout, by influencing preventive programmes adjusted by the specific features of the strategies for handling stress in the workplace [40].

The study participants were middle-aged European adults, mostly women, in a stable relationship and with high levels of education, mostly working as TRS. Most had worked at the university for between four and sixteen years; more than half were permanent employees and most of them worked full-time. Approximately one third of the participants had taken sick leave in the previous year, with a mean of almost four weeks. With regard to the scores obtained for the different scales utilized, the BCSQ-12 dimension with the lowest mean score was neglect, perhaps because of the social desirability effect, given the importance that western countries give to accomplishment at work [12]. Substance use was the COPE dimension with lowest mean score, meaning that it was the coping strategy least reported by the participants. Likewise, this result should be viewed with caution due to the well-known social desirability effect related to self-reporting of substance use or misuse [41].

The raw correlations showed that denial presented low but significant associations with all of the BCSQ-12 dimensions, so it may be present as a characteristic common to each burnout type. This commonality could be explained by the viewpoint of social exchange [5], [42], [43]: feelings of discontentment are present in all the profiles owing to the discrepancy between the personal contributions and the gratification obtained in return [6], [44]. We were therefore able to associate the denial characteristic of the general lack of acceptance observed throughout the burnout types. Acceptance is a modern construct in medicine and psychology that is related to psychological flexibility and resilience [45]–[49]. Thus, denial could be driving workers to a dysfunctional manner of coping with their daily stressors in the workplace. Venting of emotions also presented significant bivariate relationships with all of the subscales belonging to the different burnout subtypes. This coping strategy could be a general feature of burnout, although the specific content presented in the complaints could be different depending on the type of burnout experiences, as has recently been reported [50]. Consequently, the frenetic subtype would complain about the organizational hierarchy, which imposes limits to his or her high ambition; the underchallenged subtype would express distress about the routine nature of his or her obligations, which would hinder personal development; and the worn-out subtype would be annoyed by the monitoring systems, owing to his or her negligent behaviour. The perception of continuous complaints by colleagues could increase the emotional exhaustion experienced and contribute to the development of negative attitudes, creating a type of ‘contagion’ mechanism for spreading the syndrome to other colleagues in the workplace [15], [51]. Planning was significantly associated with all the BCSQ-12 dimensions, positively in the case of overload and negatively for the lack of development and neglect, the latter association being the stronger one. This result seems to support the burnout typology as a classification of the syndrome according to the commitment at work, as underlies this theoretical proposal [10]. Lastly, positive reinterpretation was directly and significantly related to overload, and inversely and significantly related to neglect. These relationships could indicate an important difference between the profiles with regard to their particularities and their contrast in engagement [26], [27].

We observed that overload was mainly explained by venting of emotions as a coping strategy. In general, the use of palliative strategies with a focus on emotions is related to physical discomfort [28]. An emotion-oriented coping style can predict emotional exhaustion and depression, and may contribute to the development and maintenance of psychological problems [26]. In fact, reductions in the use of emotion-focused coping may decrease levels of exhaustion [27]. On the other hand, as previously stated, overload was also explained by the focus on solving situations. This strategy could be responsible for maintaining high levels of efficacy in this subtype of burnout [6], [10]–[12], [52]. Actually, problem-focused coping behaviours have been associated with high demands and high levels of self-efficacy [53]. Nevertheless, the fact that venting of emotions gains greater relevance than focusing on solving situations when explaining overload alerts us to the psychological distress that this burnout profile suffers. Finally, overload was also explained by religion. This relationship can be understood if we consider effort and resignation at work to be fundamental western values. This aspect is reflected in the positive relationships between religious coping and anxiety symptoms [54], and in the tendency to rely on religious beliefs and practices in times of illness to relieve stress, retain a sense of control and maintain hope, meaning and purpose [55]. In summary, the overloaded frenetic subtype appears to be the most work-involved profile, given its coping pattern based on solving situations. The use of this strategy in an inflexible way, as a result of high ambition, could lead to exhaustion, especially if the subject blames others for his or her dysphoric feelings, using venting of emotions at the same time [56]. This burnout type would need to improve emotional regulation to contribute to the reduction of the psychopathological symptoms of burnout [26]. This strategy would not suffice, however, if it were not accompanied by increasing psychological flexibility, something that has been shown to be relevant to the treatment of burnout [56], [57], and which is underpinned by therapies such as the Acceptance and Commitment Therapy (ACT) [58].

In general terms, the neglect dimension of the worn-out type of burnout is found in the opposite situation because it carries lack of dedication. In fact, neglect was only significantly explained by behavioural disengagement, the inverse strategy of problem-focused behavioural coping; thus, it was associated with a passive coping tendency, which has been positively associated with high levels of job stress [59]. This type of coping is also positively related to all classical burnout symptoms and is negatively linked to self-efficacy and job satisfaction [19], [29]. The use of the disengagement coping strategy has been said to mediate the relationships between job stress and burnout [25], and it may be the main variable responsible for inefficacy [9], [12]. In other words, the neglected worn-out subtype is immersed in abandonment at work, using behavioural disengagement as a coping strategy. The use of this strategy may result from inconsequential histories of contingencies regarding awards and control [33] and could drive one to low performance levels through inefficacy perception [60], which may cause difficulties in alleviating stress [61]. In general, this burnout type seems to first require behavioural activation, perhaps by giving priority to commitment aspects from ACT or other types of cognitive and behavioural therapies to reduce stress and burnout symptoms [62], but also by eliminating negative burnout-related cognitions that would serve to perpetuate the syndrome [63], [64].

Lack of development from the under-challenged type of burnout was significantly explained by both venting of emotions and behavioural disengagement, which are also present in overload and neglect, respectively. Thus, lack of development seems to be in a middle position between the extremes, as it shares characteristics with the other two profiles. However, it was mainly explained by cognitive avoidance as a coping strategy, which has also been related to physical discomfort [28], so this profile of burnout would have particular features along those lines as well. In general, escapist coping strategies such as avoidance, even if used only occasionally, may be strong predictors of burnout in its classical definition [65], and may increase the use of substance abuse as a coping strategy [29]. The presence of avoidance in general [66], and experiential avoidance in particular [67], has specifically been related to depersonalisation, the corresponding cynicism dimension in the human services professions. This last one is high and directly associated with lack of development, which is likely due to a relative distancing from obligations [9], [12]. Avoidance is also related to the absence of acceptance [45]–[49], reinforcing a dysfunctional coping profile. Consequently, the development-lacking under-challenged subtype takes a step toward indifference, owing to its use of cognitive avoidance as a main coping strategy. The utilization of this strategy could raise levels of boredom and cynicism, increasing detachment from tasks as a result of distorted basic assumptions regarding success and achievement [56]. Therefore, this burnout type would benefit from developing presence at work through mindfulness or values-based therapies, both of which are included in ACT and have proven successful in the treatment of burnout [68], [69]. Moreover, this burnout type could also benefit from the use of positive reappraisal coping [70], which is absent in this profile.

The main limitation of this study is the fact that its cross-sectional design did not allow us to draw strict conclusions about the aetiology of burnout subtypes. Furthermore, the fact that the study population consisted of employees of a single university, who were ultimately self-selected, and the difference found in the response rates by type of occupation, reduced the possibilities for generalising our results. We also observed that grant holders were more participative than others and were therefore over-represented, which can be explained by their reduced tendency to show neglect [13]. In general, as we did not have sociodemographic data from the total reference population, it was not possible to contrast to what extent the obtained sample was representative of it. In addition, the fact that measurements of stress in the workplace were not taken partly hindered the interpretation of our results. The study also has many strengths: it was carried out with a random, broad and multi-occupational sample of employees in at-risk occupations with face-to-face personal contacts [15]. Additionally, the fit of the regression models was adequate. The distribution of residuals was normal, and no autocorrelation or collinearity problems were detected, so the basic assumptions for the type of data analysis utilised were accepted. Finally, data quality was controlled by eliminating possible errors in the questionnaire transcription process through the use of purpose-designed software.

## Conclusions

Our findings support the hypothesis that different coping styles are associated with the diverse burnout subtypes. Overload was explained mainly by venting of emotions, although it was also explained by a focus on solving situations and religion; lack of development was explained mainly by cognitive avoidance, but it was also explained by venting of emotions and behavioural disengagement; neglect was explained only by behavioural disengagement. In general, a progressive decrease in levels of engagement is understood to be the response adopted by workers experiencing burnout in order to cope with stress and frustration [9]. This aspect seems to be an important factor in explaining the differences between the subtypes from a longitudinal perspective [6], [10]–[12], and could be the keystone for developing new treatment interventions adjusted to the coping strategies of each particular case. Cognitive and behavioural therapies, such as ACT, may be useful for all burnout types, emphasising the different modules according to the degree of dedication at work. However, this therapeutic model is hypothetical and its effectiveness must be evaluated.
